# Impaired Reproductive Development in Sons of Women Occupationally Exposed to Pesticides during Pregnancy

**DOI:** 10.1289/ehp.10790

**Published:** 2008-01-22

**Authors:** Helle R. Andersen, Ida M. Schmidt, Philippe Grandjean, Tina K. Jensen, Esben Budtz-Jørgensen, Mia B. Kjærstad, Jesper Bælum, Jesper B. Nielsen, Niels E. Skakkebæk, Katharina M. Main

**Affiliations:** 1 Institute of Public Health, Department of Environmental Medicine, University of Southern Denmark, Odense, Denmark; 2 University Department of Growth and Reproduction, Rigshospitalet, Copenhagen, Denmark; 3 Department of Biostatistics, University of Copenhagen, Copenhagen, Denmark; 4 Department of Occupational and Environmental Medicine, Odense University Hospital, Odense, Denmark

**Keywords:** cryptorchidism, greenhouse workers, hormones, occupational exposure, pesticides, prenatal, reproductive development

## Abstract

**Objectives:**

The aim of this prospective study was to investigate whether occupational pesticide exposure during pregnancy causes adverse effects on the reproductive development in the male infants.

**Design and measurements:**

Pregnant women employed in greenhouses in Denmark were consecutively recruited, and 113 mother–son pairs were included. The mothers were categorized as occupationally exposed (91 sons) or unexposed (22 sons) to pesticides during pregnancy. Testicular position and volume, penile length, and position of urethral opening were determined at 3 months of age using standardized techniques. Concentrations of reproductive hormones in serum from the boys were analyzed.

**Results:**

The prevalence of cryptorchidism at 3 months of age was 6.2% [95% confidence interval (CI), 3.0–12.4]. This prevalence was considerably higher than among Danish boys born in the Copenhagen area (1.9%; 95% CI, 1.2–3.0) examined by the same procedure. Boys of pesticide-exposed mothers showed decreased penile length, testicular volume, serum concentrations of testosterone, and inhibin B. Serum concentrations of sex hormone-binding globulin, follicle-stimulating hormone, and the luteinizing hormone:testosterone ratio were increased compared with boys of nonexposed mothers. For individual parameters, only the decreased penile length was statistically significant (*p* = 0.04). However, all observed effects were in the anticipated direction, and a joint multivariate test showed that this finding had a *p*-value of 0.012.

**Conclusions:**

Our findings suggest an adverse effect of maternal occupational pesticide exposure on reproductive development in the sons despite current greenhouse safeguards and special measures to protect pregnant women.

Sex hormones are involved in the regulation of fetal sex differentiation, and disturbance of the hormonal balance at critical developmental stages can affect the phenotype and gonadal function (Sharpe 2006a). Fetal exposure to manufactured substances with endocrine-disrupting effects is a potential cause of cryptorchidism and related disorders in the male reproductive system (Skakkebæk 2002; Skakkebæk et al. 2001). Geographic differences and adverse temporal trends in male reproductive health in humans have been reported in several studies (Adami et al. 1994; Auger et al. 1995; Carlsen et al. 1992; Jorgensen et al. 2002). To investigate and compare male reproductive development among Nordic countries, The Nordic Cryptorchidism Study Group was established. Among recent findings, higher prevalence of cryptorchidism and lower serum concentrations of inhibin B, follicle-stimulating hormone (FSH), and sex hormone–binding globulin (SHBG) and smaller testicular volumes at 3 months of age were recently reported for Danish boys, compared with Finnish boys (Main et al. 2006b). As a part of these studies, a cohort of Danish boys was established in the Copenhagen area, and standardized examination procedures for classification of testicular position and hypospadias (Boisen et al. 2005), testicular size determined by ultrasound (Main et al. 2006b), and penile length (Boas et al. 2006) were developed.

The role of exposure to endocrinedisrupting chemicals as explanation for the observed differences in reproductive health is unclear, although the evidence for such an association is strong from studies in wildlife and laboratory animals (Damstra et al. 2002; Sharpe and Irvine 2004). A considerable number of the chemicals identified as endocrine disruptors are pesticides (Andersen et al. 2002; Kojima et al. 2004; Vinggaard et al. 2000), and increased prevalence of cryptorchidism has been reported in sons of women working as gardeners (Weidner et al. 1998) or living on farms where pesticides have been used (Kristensen et al. 1997). A similar tendency was seen for mothers working in agriculture and exposed to pesticides (Carbone et al. 2007). Additionally, a higher prevalence of cryptorchidism and hypospadias (Carbone et al. 2006) and a higher rate of orchidopexy (surgical treatment of undescended testicles) (Garcia-Rodriguez et al. 1996) were found in areas with extensive farming and pesticide use. However, none of these studies obtained individual exposure assessments. Recently, an association was reported between cryptorchidism and the content of some persistent pesticides in maternal breast milk (Damgaard et al. 2006).

In temperate climates, the highest occupational pesticide exposure likely occurs in greenhouses—especially in the production of ornamental plants and cut flowers—that involve inadequate ventilation, frequent application of pesticides, and manual handling of the treated plants (Brouwer et al. 1992; Illing 1997). Many women in fertile age groups are employed in this industry, with a total of approximately 2,500 women in Denmark. We hypothesized that women who become pregnant while working in greenhouses where pesticides are applied have an increased risk of giving birth to a boy with abnormal development of the reproductive organs. In the first months of life the hypothalamus–pituitary–gonadal axis in male infants is briefly activated, and this period is thought to be a useful diagnostic window for signs of hypogonadism and a harbinger of adult reproductive dysfunction (Grumbach 2005; Main et al. 2000, 2006b; Suomi et al. 2006). Thus, it is possible to assess reproductive development in close temporal relation to the suspected *in utero* exposure. We therefore initiated a prospective cohort study to investigate possible associations between occupational pesticide exposure in greenhouses during pregnancy and reproductive development of the children. We used the standardized examination procedures developed by The Nordic Cryptorchidism Study Group, and all examinations were performed by one pediatrician, who also examined most of the boys born in the Copenhagen area.

## Materials and Methods

### Study design

#### Pregnant women

**Figure 1:**
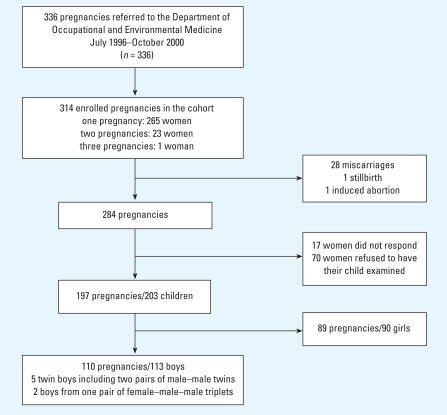
Total number of enrolled pregnancies and the number of dropouts.

Pregnant women employed in greenhouses in Funen, Denmark, were recruited consecutively from July 1996 to October 2000 at the Department of Occupational and Environmental Medicine at Odense University Hospital. A previous survey (unpublished), suggested that approximately 50% of all pregnant women working in greenhouses in Funen are referred to this department for advice regarding their working conditions during pregnancy. In an attempt to increase the percentage of referrals, an information letter was sent to all general practitioners and owners of greenhouse settings. After oral and written information about the study, 289 women agreed to participate and signed a written consent. Their 314 pregnancies correspond to 93% of a total of 336 pregnancies among greenhouse workers referred to the department. One woman was included with three separate pregnancies, and 23 women participated with two separate pregnancies (Figure 1).

The study was conducted according to the Helsinki II Declaration (World Medical Association 2000) with written informed consent by all mothers and was approved by the regional Danish ethical review committee and the Danish Data Protection Agency.

#### Exposure information

In Denmark, pregnant women have a legal right to paid leave if the working situation is considered a risk for the pregnancy outcome. Risk assessments are offered free of charge by the governmental health care system. For pregnant women included in the present study, individual assessment of working conditions was performed at the Department of Occupational and Environmental Medicine, Odense University Hospital, by a physician certified in occupational medicine in cooperation with a toxicologist, and was independent of the study protocol as such. The employers were contacted by telephone to obtain detailed information about working conditions, especially regarding recent and scheduled use of pesticides (trade names and spraying frequency). If pesticides were used, the employer was asked whether it was possible to move the woman to work functions without pesticide exposure or to adjust the working conditions to obey prolonged time intervals for reentry and handling of treated plants. Required reentry intervals for each pesticide were set by the toxicologist based on information on toxicity and degradation time for the pesticides. A letter with the established time intervals was sent to the employer. If the requirements could be met, if the woman could be moved to pesticide-free areas, or if no pesticides were used, the woman was advised to continue work. Most women recommended for paid leave were employed in companies with rather high pesticide use or in small companies with less possibility for rotation of work or special protective measures.

Before the consultation, pregnant women were informed in writing about the study. At the end of the consultation, those women who agreed to participate in the study answered a more detailed questionnaire-assisted interview regarding the following:

- Working conditions (i.e., job function, personal handling of pesticides, names and application frequency of pesticide products, reentry intervals for entering greenhouses where pesticides had been applied, procedures for handling of plant cultures recently treated with pesticides, and use of protective equipment)
- Reproductive history (current and previous pregnancies, use of contraceptives, waiting time to pregnancy) as well as information about age and general health parameters for the woman and her partner
- Lifestyle and social factors including educational background, smoking and drinking habits, nonoccupational exposure to pesticides (home use for pets or gardening), and occupation of her partner.

For all women, reentry activities (such as moving or packing pot plants or nipping cuttings) constituted the main work functions. Besides, 50 (17%) of the women reported they had been directly involved in applying pesticides, mainly by irrigating fungicides or growth retardants. Approximately 200 different pesticide formulations, representing 124 different active pesticide ingredients, were used in the working areas. Some of the pesticides were used only in a few greenhouses or during restricted time periods, whereas others were used more frequently. None were used in all greenhouses. The active ingredients used most frequently were the insecticides deltamethrin, dichlorvos, dimethoate chlorpyrifos, endosulfan, fenpropathrin, fipronil imidacloprid, methiocarb, methomyl, and pirimicarb; the fungicides captan, chlorothalonil, fenarimol, fosetyl-aluminium, iprodion, prochloraz, propamocarb tolchlofosmethyl, and vinclozolin; and the growth regulators daminozid, paclobutrazol, chlormequat chlorid, and ethephon. A complete list of pesticides used in the greenhouses can be obtained from the corresponding author.

Although we have detailed information about working conditions and pesticide use, the lack of knowledge about endocrine-disrupting properties for most of the pesticides hampered the exposure evaluation. Besides, individual awareness and behavior (e.g., frequency of hand washing, correct use of gloves and other protective equipment) may have changed after recognition of pregnancy and interview. Hence, the women were categorized as occupationally exposed if pesticides were applied in the working area more than once a month and/or the women handled treated plants within 1 week after treatment and/or the women were directly involved in applying pesticides. The women were categorized as occupationally unexposed (controls) if none of the above criteria was fulfilled. Most of the women categorized as unexposed worked in the production of tomatoes, cucumbers, or cactuses, where chemical pesticides had been replaced with biological pest control, or in separate greenhouses of other horticultures where pesticides were never or very seldom (once a month or less) used. One of the unexposed women had office work, and two women had not been at work for several months before conception because they were on educational leave. Classification of the mothers as pesticide exposed or unexposed controls was done independently by two toxicologists with special expertise in working conditions in greenhouse horticultures, and was performed before the results from examination of the children became available.

#### Children

During gestational week (GW) 24, a questionnaire was mailed to all women to obtain updated information about pregnancy and working conditions as well as estimated term and place of parturition. Two weeks after the expected time of childbirth, a request was sent to the hospital to obtain obstetric records. If the pregnancy outcome was a live birth, a letter was sent to the mother inviting her to have the child examined at approximately 3 months of age, adjusted for gestational age.

Of 314 pregnancies included in the study, 28 (8.9%) were miscarriages, 1 was an induced abortion, 1 was a stillbirth, and 284 (90.4%) pregnancies resulted in ≥ 1 live-born children (Figure 1). Infants from 197 (62.7%) pregnancies were examined. The remaining 87 mothers dropped out of the study. Sixty-nine mothers responded that they did not want to have their child examined, one family had moved out of the area, and 17 mothers did not reply to letters and could not be reached by telephone. A higher percentage of those women who dropped out of the study were of non-Danish ethnicity or were not recommended to have paid leave during their pregnancy (Table 1). Three of the women who were not recommended to have paid leave stated that they were disappointed about the decision and therefore refused to have their child examined. This explanation may apply to more of the women than those who directly stated as much. Four mothers did not want their child examined because of the blood sampling, and one mother withdrew consent because her child underwent surgical treatment for clubfoot.

A total of 203 infants (113 boys and 90 girls) were examined at a mean age of 3.18 months after the expected date of delivery (range, 2.33–5.43 months of age). Among them was one set of triplets, four sets of twins, and 10 sets of siblings. This paper presents the observations in the boys.

### Clinical examination of the boys

The examination followed the same standardized procedure as used by The Nordic Cryptorchidism Study Group. Detailed description of methods are given in previous publications: classification of testicular position (Boisen et al. 2004) and hypospadias (Boisen et al. 2005), testicular size determined by ultrasound (Main et al. 2006b), and penile length (Boas et al. 2006). Children were considered small for gestational age when age-adjusted birth weight was > 22% below the mean (equivalent to < –2 SDs) of a sex-differentiated reference group (Larsen 2001). The examinations were performed blinded to the pesticide exposure level of the mothers by a single pediatrician involved in the studies cited.

### Hormone assays

Venous nonfasting blood samples were obtained from 85 (75%) of the boys at the examination. Only one attempt at venipuncture was carried out, which was approved by the ethical review committee. Serum was stored at –20°C until analysis. Serum concentrations of FSH, luteinizing hormone (LH), SHBG, testosterone, and inhibin B were measured in coded samples at the laboratory at the University Department of Growth and Reproduction at Rigshospitalet in Copenhagen by methods as previously described (Boisen et al. 2005). The serum concentration of LH was used only for calculation of the LH:testosterone ratio. This ratio was considered to be a more reliable marker of testicular function in the boys than the serum LH concentration because the postnatal peak of serum concentrations of LH and testosterone is relatively brief (Andersson et al. 1998) and the timing of blood sampling varied between 2.33 and 5.43 months of age.

### Statistical analysis

We tested difference in prevalence of cryptorchidism between sons of greenhouse workers and boys from the Copenhagen cohort by chi-square test. The crude relative risk and 95% confidence interval (CI) were calculated.

For the sons of greenhouse workers, differences in characteristics of parents and birth information between the group of cryptorchid and noncryptorchid boys and between the group of boys whose mothers were occupationally exposed to pesticides and unexposed were tested by Mann–Whitney *U*-test (continuous data) or Fisher’s exact test (numeric data).

We first performed age-adjusted partial correlation analyses to establish the associations between testicular volume, penile length, and reproductive hormones after transformations, as described below. We estimated differences in penile length, testicular volume, and reproductive hormones between the group of boys prenatally exposed to pesticides and unexposed by standard multiple regression analysis with confounder adjustment. A uniform set of continuous covariates consisting of birth weight, birth length, gestational age, age at examination, and two proxy covariates—small for gestational age class and smoking during pregnancy—was used for all outcomes. Any influential points were identified, and residual plots were used to check the model fit. When the assumptions of the model seemed not to be satisfied, transformation of the outcome variables was considered. Although penile length and SHBG showed a satisfactory fit of the multiple regression model, a logarithmic transformation was required for testicular volume, testosterone, FSH, inhibin B, and the LH:testosterone ratio. Confounder-adjusted effects were expressed as the mean difference, whereas the relative difference (in percent) was calculated for log-transformed outcomes. Because hormonal changes have been reported for them (Main et al. 2006a; Suomi et al. 2006), cryptorchid boys were omitted from these statistical analysis.

According to the study hypothesis, an adverse effect of prenatal exposure to endocrine-disrupting pesticides would lead to a decrease in penile length, testicular volume, and serum testosterone and inhibin B concentrations, and increased serum concentrations of SHBG, FSH, and the LH:testosterone ratio. The standard univariate *p*-values evaluate the significance of each group difference separately and do not take into account that all observed effects were in the anticipated direction. We therefore conducted a multivariate test assessing the likelihood that the observed structure in the group differences could have arisen by chance. Specifically, we tested whether all six group differences were zero against the one-sided multivariate alternative that at least one of the effects was present and in the direction expected (Kudo 1963). We used a likelihood ratio test, which allowed for the correlation between the outcomes. To avoid relying on asymptotic theory, the *p*-value was determined by Monte Carlo simulations. This multivariate test provides an overall assessment of the observed tendencies in the data and is not affected by the multiple testing problems associated with the standard univariate testing procedures.

## Results

At 3 months of age, 7 of the 113 boys had undescended testicles. One cryptorchid boy was a twin born with a brother with normal testicles. Another cryptorchid boy had a brother with normal testicles included in the study 2 years before. The remaining five boys were first-born singletons. One boy had bilateral nonpalpable testicles (normal male genotype was confirmed), and one had bilateral high scrotal testicles. The remaining five boys had unilateral cryptorchidism. No case of hypospadias was observed. None of the cryptorchid boys had a birth weight < 2,500 g or was born before GW37 (Table 2).

There was no significant difference (*p* = 0.34) in prevalence of cryptorchidism between boys born of exposed mothers and unexposed mothers, although all mothers of boys with cryptorchidism were exposed to pesticides (Table 3). One of these mothers was released from working procedures involving pesticides beginning at GW10, one was fired and left the workplace in GW4, and five had paid leave and left the workplace between GW3 and GW9.

The prevalence of congenital cryptorchidism among the sons of greenhouse workers in this study was 6.2% (95% CI, 3.0–12.4). This prevalence was significantly higher than among boys born in the Copenhagen area (Boisen et al. 2004) (Table 4). The prevalence among the 91 sons of pesticide-exposed mothers was 7.7% (95% CI, 3.7–15.3).

After omission of the seven cryptorchid boys and controlling for age at examination, serum FSH was significantly negatively correlated to the serum inhibin B concentration (*r* = –0.43, *p* < 0.0001) and positively to the LH:testosterone ratio (*r* = 0.32, *p* = 0.01). Testicular volume was positively correlated to penile length (*r* = 0.12; *p* = 0.26) and testosterone (*r* = 0.15, *p* = 0.22), and negatively correlated to FSH (*r* = –0.15, *p* = 0.24) and the LH:testosterone ratio (*r* = –0.25, *p* = 0.04). Penile length was positively correlated to testosterone (*r* = 0.14, *p* = 0.25) and negatively correlated to the LH:testosterone ratio (*r* = –0.15, *p* = 0.22).

The group of boys whose mothers were exposed to pesticides had decreased penile length, testicular volume, serum concentrations of testosterone, and inhibin B, whereas serum concentrations of SHBG and FSH as well as the LH:testosterone ratio were increased (Table 5). When analyzed separately, only the decreased penile length was statistically significant (*p* = 0.04). However, for all outcomes the exposure effect was in the anticipated direction. The combined results were then entered into a joint multivariate test, which showed a *p*-value of 0.012.

## Discussion

Female greenhouse workers with confirmed pesticide exposure during pregnancy gave birth to boys with smaller penises and testicles, lower serum concentrations of testosterone and inhibin B, higher serum concentrations of SHBG and FSH, and higher LH:testosterone ratio than unexposed workers. These results suggest an adverse effect of pesticides on Leydig and Sertoli cells during testicular development. In addition, female greenhouse workers had a more than three-fold increased risk of delivering a boy with cryptorchidism compared with women from the urban area of Copenhagen.

In regard to covariates of possible importance, there was no difference in median gestational age between cryptorchid and noncryptorchid boys, and none of the cryptorchid boys was born preterm. Although the median birth weight was slightly lower in the cryptorchid boys, none of these boys had a birth weight < 2,500 g, and only one of the cryptorchid boys was small for gestational age. Hence, differences in distribution of known risk factors for cryptorchidism (low birth weight, small for gestational age, preterm delivery) (Akre et al. 1999; Aschim et al. 2004; Weidner et al. 1999) cannot explain the increased number of cryptorchid boys born of female greenhouse workers compared with the Copenhagen cohort. Maternal smoking during pregnancy has been associated with increased risk of cryptorchidism in a few studies (Jensen et al. 2007; Thorup et al. 2006) but not in others (Biggs et al. 2002; Moller and Skakkebæk 1996). However, the percentage of smokers was similar in the greenhouse worker cohort (36.3%) and the Copenhagen cohort (35.5%) (data not shown). A recent study found an association between maternal alcohol consumption and risk of cryptorchidism (Damgaard et al. 2007). Among the greenhouse workers, 34.8% reported weekly alcohol consumption (1–5 drinks per week), compared with 48.8% in the Copenhagen cohort. Hence, differences in smoking and alcohol habits are unlikely to explain the difference in prevalence of cryptorchidism between the two cohorts.

Among the strengths of this study, all the boys were examined by the same pediatrician, who also examined most of the boys in the Copenhagen cohort, and exactly the same standardized procedures were followed to ascertain cryptorchidism. To avoid information bias, the pediatrician did not have access to any information about possible pesticide exposure of the mother during pregnancy before the examination, and the mother’s working conditions were not discussed during the examination. Classification of the mothers as pesticide exposed or unexposed was performed independently by two toxicologists with special expertise in working conditions in greenhouse horticultures, and the risk of misclassification is considered low. This classification was completed before the children were examined. The small number of unexposed controls is a limitation of the study, because it diminishes the possibility to detect differences between exposed and unexposed. In addition, the women categorized as unexposed controls may still be more exposed than non-greenhouse workers, thereby possibly causing an underestimation of the exposure-associated risk. A high fraction (40%) of the pregnant women left the workplace for paid leave early in pregnancy. This preventive measure may have reduced the likelihood of adverse pesticide effects, also leading to an underestimation of the risk.

Estimation of the individual exposure to endocrine-disrupting pesticides was not possible because endocrine-disrupting properties and dermal uptake rates were unknown for most of the pesticides used in the working areas. Blood samples were obtained from the mothers at enrollment, and a biomarker of xenoestrogenic activity in serum demonstrated an exposure-associated increase (Andersen et al. 2007). However, the association between pesticide exposure and xenoestrogenic activity was statistically significant only for women who had been at work within the last week before blood sampling. Because approximately half of the women examined in the present study had not been at work for greater intervals of time before blood sampling, this assay could not be used to estimate the xenoestrogen exposure level.

A possible weakness of this study is that a relatively large number of women (*n* = 87) dropped out of the study after enrollment. In general, the exposure level was lower among those who dropped out, as indicated by a higher percentage categorized as unexposed, a lower percentage of women applying pesticides, and a lower percentage recommended to have leave from work (Table 1). Hence, unexposed and minimally exposed women might have been less motivated to participate in the study, thereby possibly introducing selection bias toward those with higher pesticide exposure. However, the work functions of all women in this study were very similar, and it seems unlikely that a slightly lower pesticide exposure among the 65 pesticide-exposed women who dropped out of the study would have changed the results markedly. The remaining 22 women who dropped out were categorized as unexposed and hence were lost for the control group. There were no differences in age or smoking behavior between the women who dropped out and those who had their child examined. For 26 families, the decision not to have the child examined was taken before delivery and was stated in the completed questionnaire from GW24. Besides, selection bias is not likely to be a major problem for comparisons within a study population of both exposed and unexposed greenhouse workers.

Our findings are supported by previously published evidence. In an Italian case–control study (Carbone et al. 2007), cases of cryptorchidism were identified via records in the local pediatric service and confirmed by a surgical consultant. Although not statistically significant, a clear tendency was observed toward increased risk of cryptorchidism among sons of women working in agriculture and exposed to pesticides (Carbone et al. 2007). An increased risk of cryptorchidism was also reported in boys born on Norwegian farms where pesticides were applied (Kristensen et al. 1997). In a previous Danish register-based case–control study, an increased risk of cryptorchidism was found in sons of women working in gardening (Weidner et al. 1998). Most cases were identified by records of surgical treatment of cryptorchidism in the Danish National Patient Register, and additional cases were identified in the Danish Malformation Register (Weidner et al. 1998). However, a recent study found no increased risk of cryptorchidism among boys from the Danish National Birth Cohort born of women working as gardeners or farmers (Zhu et al. 2006). In the latter study, information about cryptorchidism were obtained only by linkage to the Danish Malformation Register that is likely to underreport these birth defects (Toppari et al. 2001). Especially mild and transient forms of cryptorchidism, which are much more frequent at birth than severe forms of cryptorchidism (Damgaard et al. 2006), are incompletely reported to malformation registers. However, boys with transient cryptorchidism also show signs of subtle impairment of testicular function (Suomi et al. 2006). Previous studies have not included the milder forms of cryptorchidism, although identified through the detailed examinations employed in the present study and in the Copenhagen cohort.

A prevalence of hypospadias at 3 months of age was found to be 1.03% among Danish live-born boys (Boisen et al. 2005) using the same examination procedure as in this study. Hence, our study population of 113 boys was too small to identify minor changes in hypospadias rates. Only one previous study has so far demonstrated an association between parental pesticide exposure and increased risk of hypospadias in the sons (Kristensen et al. 1997).

Fourteen of 21 pesticides, selected as the most frequently used in the working areas of the pregnant women, possessed endocrine-disrupting potential in one or more cellular assays (Andersen et al. 2002), thus indicating that a considerable number of the remaining pesticides may have similar properties. Three fungicides used often in the greenhouses—fenarimol, vinclozolin, and prochloraz—have also been demonstrated to be endocrine disruptors in animal studies (Andersen et al. 2006; Gray et al. 1999; Vinggaard et al. 2002, 2005b), and prochloraz and vinclozolin disturbed sexual differentiation in male rats after prenatal exposure (Gray et al. 1999; Laier et al. 2006; Noriega et al. 2005; Shono et al. 2004; Vinggaard et al. 2005a).

All pesticides used in the working areas were currently approved pesticides with low biological persistence. The longest excretion times reported from animal studies for these pesticides were up to a few weeks and in most cases no more than a few days. Hence, the effects observed are most likely induced during the period where the mother was exposed (or shortly after) and not later in pregnancy, or during breast-feeding, as may be the case for persistent pesticides (Damgaard et al. 2006).

Because all mothers of cryptorchid boys were removed from occupational pesticide exposure between GW3 and GW10, any effects must have been initiated early in pregnancy. The first weeks of gestation cover the most critical window for testicular determination in humans (Virtanen et al. 2007). Testicular descent is divided into a transabdominal and a transinguinal phase, which appear to be intricately regulated although not completely understood in humans. Testicular hormone production plays an essential role for the normal testicular descent. From GW6, anti-Müllerian hormone produced by Sertoli cells stimulates regression of Müllerian ducts in male fetuses, and at the same time, the first appearance of Leydig cells and subsequent testosterone production occur (O’Shaughnessy et al. 2006; Sizonenko 1993). In addition to ensuring masculinization, the fetal Leydig cells act to induce the first, transabdominal phase of descent of the testicles through secretion of insulin-like factor 3 (Insl-3) (Klonisch et al. 2004). Early interference with testicular development and differentiation will subsequently impair primary testicular function and, as a result, testicular descent (Sharpe 2006b; Skakkebæk et al. 2001).

Leydig cell proliferation, germ cell differentiation, and the replication of Sertoli cells during the postnatal phase are closely interlinked processes (Main et al. 2006a). Therefore, cross-correlations between the two testicular hormones (testosterone and inhibin B) and the two gonadotropins can be detected during the brief postnatal activation of the hypothalamus–pituitary–testicular axis. Hormonal regulation in newborn boys appears to be similar to the negative feedback observed in puberty and onward (Andersson et al. 1998; Main et al. 2006b; Suomi et al. 2006). Hence, disturbance of Sertoli cell function would be expected to lead to a decrease of the serum concentration of inhibin B and an increase of the FSH level due to negative feedback. Accordingly, inhibin B was reported to be negatively correlated to FSH at 3 months of age (Main et al. 2006b), as also reflected by the results in our study. In addition, serum FSH at 3 months of age has been reported to be higher in boys with hypospadias (Boisen et al. 2005) or cryptorchidism (Suomi et al. 2006) than in normal boys as an indicator of primary testicular dysfunction. Impairment of Leydig cell function would be expected to cause decreased Insl-3 and testosterone production and a resulting stimulation of LH release from the pituitary gland, thereby causing an increase in the LH:testosterone ratio. The serum concentration of SHBG is regulated by numerous hormones including androgens (Toscano et al. 1992), and during infancy there is a negative feedback between serum SHBG levels and serum testosterone (Belgorosky and Rivarola 1985). Thus, serum SHBG will be increased at lowered androgen levels.

In the present study, the distribution of all reproductive parameters between the exposed and unexposed group supported our hypothesis that prenatal exposure to currently used pesticides may adversely affect testicular development in male infants. The joint multivariate analysis strongly indicated a true association. Because the seven cryptorchid boys, who all had exposed mothers, were omitted from the multivariate analysis, the observed differences cannot be explained by altered hormone concentrations and impaired gonadal development in cryptorchid boys (Main et al. 2006a; Suomi et al. 2006). Although the observed effects are subtle on an individual level, the biological link between them (i.e., decreased androgen levels contributing to cryptorchidism, reduced penile length, reduced testicular volume, and increased gonadotropin levels) should raise concern about effects at population levels. The consequences of the effects observed for fertility and testicular function in adulthood are unknown. However, lower serum concentrations of inhibin B, FSH, and SHBG and smaller testicular volumes at 3 months of age were recently reported for Danish boys compared with Finnish boys (Main et al. 2006b). These differences are thought to be related to the differences between the two countries in sperm counts and incidence of testicular cancer (Adami et al. 1994; Jorgensen et al. 2002).

In conclusion, this study showed that female greenhouse workers had increased risk of delivering a boy with cryptorchidism and that pesticide-exposed greenhouse workers had boys with smaller penises and testicles, lower serum concentrations of testosterone and inhibin B, higher serum concentrations of SHBG and FSH, and higher LH:testosterone ratio than unexposed workers. Thus, the results suggest an adverse effect of maternal occupational pesticide exposure on reproductive development in the sons, despite the exposures occurring within highly controlled greenhouse operations and special measures to protect pregnant women. Accordingly, workers, especially young women, should be protected against pesticide exposure by enforcing longer reentry intervals and more comprehensive use of protective equipment.

## Figures and Tables

**Table 1:** Characteristics of women who agreed to have their child examined at 3 months and women who dropped out of the study.

Characteristic	Child examined	Families did not want the child examined	No response
No. of pregnancies	197	70	17
Maternal age, mean (range)	27.4 (19.0–36.6)	27.1 (18.9–40.1)	26.4 (18.3–33.2)
Maternal smoking [no. (%)]	61 (31.0)	21 (30.0)	9 (52.9)
Non-Danish ethnicity [no. (%)]	12 (6.1)	13 (18.6)	2 (11.8)
Pesticide exposed [no. (%)]	162 (82.2)	49 (70.0)	16 (94.1)
Applying pesticides [no. (%)]	42 (21.3)	5 (7.4)	3 (17.6)
Leave recommended [no. (%)]	79 (40.1)	17 (24.3)	2 (11.8)

**Table 2:** Characteristics of parents and birth information for cryptorchid and noncryptorchid boys whose mothers worked in greenhouses during all or part of their pregnancies.

Characteristic	Cryptorchid boys (*n* = 7)	Unaffected boys (*n* = 106)	*p*-Value^a^
Maternal age (years)	27.7 (22.2–28.2)	28.2 (19.6–37.2)	0.26
Paternal age (years)	27 (23–32)	29 (22–43)	0.20
Age of child at examination (months)	3.09 (2.30–3.55)	3.07 (1.94–4.83)	0.59
Gestational age (days)	280 (261–291)	281 (235–297)	0.39
Birth weight (g)	3,400 (2,500–4,250)	3,654 (2,083–4,755)	0.14
Birth length (cm)	52 (47–55)	53 (47–58)	0.10
Small for gestational age [no. (%)]	1 (14.3)	6 (5.7)	0.37
Primiparous [no. (%)]	6 (85.7)	78 (73.6)	0.68
Multiple outcome [no. (%)]	1 (14.3)	3 (2.8)	0.23
Maternal smoking [no. (%)]	4 (57.1)	37 (34.9)	0.25
Maternal alcohol consumption [no. (%)]	2 (28.6)	37 (34.9)	1.00
Maternal occupational pesticide exposure [no. (%)]	7 (100)	84 (79.2)	0.34
Nonoccupational pesticide use [no. (%)]	1 (14.3)	23 (21.7)	1.00
Paternal occupational pesticide exposure [no. (%)]	0 (0)	21 (19.8)	0.35
Mother of non-Danish ethnicity [no. (%)]	0 (0)	9 (8.5)	1.00

For continuous variables, data represent median (range).

a Differences between groups were tested with Mann–Whitney *U*-test (continuous data) or Fisher’s exact test (numeric data).

**Table 3:** Characteristics of parents and birth information for boys whose mothers were occupationally exposed to pesticides during all or part of their pregnancies compared with boys whose mothers were occupationally unexposed to pesticides.

Characteristic	Unexposed	Exposed	*p*-Value^a^
No. of pregnancies/boys examined	22/22	88/91	
Maternal age (years)	28.3 (19.6–35.2)	27.7 (21.1–37.2)	0.70
Paternal age (years)	27.5 (23–40)	29 (22–43)	0.47
Gestational age at birth (days)	282 (263–297)	282 (235–297)	0.90
Birth weight (g)	3,843 (2,600–4,600)	3,600 (2,100–4,755)	0.41
Birth length (cm)	54 (48–57)	53 (47–58)	0.35
Maternal smoking [no. (%)]	10 (45.5)	29 (33.0)	0.33
Maternal alcohol consumption [no. (%)]	7 (31.8)	32 (35.6)	0.81
Mother of non-Danish ethnicity [no. (%)]	3 (13.6)	6 (6.8)	0.37
Nonoccupational pesticide use [no. (%)]	8 (36.4)	16 (18.2)	0.08
Paternal occupational pesticide exposure [no. (%)]	5 (22.7)	16 (18.2)	0.77
Primiparous [no. (%)]	17 (77.3)	67 (76.1)	1.00
Small for gestational age [no. (%)]	0 (0)	7 (7.7)	0.34
Number cryptorchid [no. (%)]	0 (0)	7 (7.7)	0.34

For continuous variables, data represent median (range).

a Differences between groups were tested with Mann-Whitney *U*-test (continuous data) or Fisher’s exact test (numerical data).

**Table 4:** Prevalence of congenital cryptorchidism at 3 months of age in sons of female greenhouse workers in Funen and boys born in the Copenhagen area.^a^

	Prevalence [% (*n*)] of congenital cryptorchidism at 3 months of age		Relative risk (95% CI)
Subcategories	Sons of greenhouse workers in Funen (*n* = 113)	Boys born in the Copenhagen area (*n* = 982)	Funen vs. Copenhagen (unadjusted)
Nonpalpable	1.8 (2)	0.2 (2)	
Inguinal	—	0.2 (2)	
Suprascrotal	0.9 (1)	0.6 (6)	
High scrotal	3.5 (4)	0.9 (9)	
Total number	6.2 (7)	1.9 (19)	3.2 (1.4–7.4)

a Danish boys from the Nordic Cryptorchidism Study (Boisen et al. 2004).

**Table 5:** Confounder adjusted values for penile length, testicular volume, and serum concentrations of reproductive hormones at three months of age in boys^a^ whose mothers worked in greenhouses during all or part of her pregnancy.

	Mean^b^(95% CI)			
	Unexposed	Exposed	B^c^ (95% CI)	*p*-Value
*N*_boys_/*N*_blood_ sample analyzed	22/14	84/61		
Penile length (cm)	4.15 (3.90 to 4.40)	3.87 (3.72 to 4.01)	–0.28 (–0.54 to –0.02)	0.04
Testicular volume^d^ (mm^3^), log transformed	178 (140 to 226)	149 (131 to 170)	–16.3 (–34.6 to 7.25)	0.16
SHBG (nmol/L)	142 (121 to162)	145 (133 to 156)	2.94 (–19.1 to 24.9)	0.79
Testosterone (nmol/L), log transformed	4.09 (3.07 to 5.45)	3.32 (2.82 to 3.91)	–18.8 (–40.1 to 9.97)	0.18
FSH (IU), log transformed	1.25 (0.96 to 1.64)	1.44 (1.23 to 1.68)	14.8 (–13.9 to 53.1)	0.34
Inhibin B (pg/mL), log transformed	370 (316 to 433)	343 (313 to 375)	–7.38 (–21.8 to 9.74)	0.37
Ratio LH/testosterone, log transformed	0.51 (0.36 to 0.72)	0.59 (0.48 to 0.72)	16.4 (–20.3 to 69.8)	0.43

*N*_boys_/*N*_blood_ samples: number of boys examined/number of blood samples analyzed. Blood samples were obtained from 16 boys in the control group and 63 boys in the exposed group, but two samples from each group were excluded from analyses because of haemolysis.

a Cryptorchid boys omitted.

b Mean (or geometric mean for log-transformed outcomes) adjusted to a median level of birth weight (3.6 kg), birth length (53 cm), gestational age (281 days), age at examination (3.09 months), appropriate weight for gestational age, and a nonsmoking mother.

c B expresses the mean difference for penile length and SHBG and the relative difference (in percent) for the log-transformed hormones and testicular volume.

d Nine boys were excluded because of insufficient ultrasound scanning.
